# Regulation of the Stress-Activated Degradation of Mitochondrial Respiratory Complexes in Yeast

**DOI:** 10.3389/fmicb.2018.00106

**Published:** 2018-01-30

**Authors:** Alba Timón-Gómez, David Sanfeliu-Redondo, Amparo Pascual-Ahuir, Markus Proft

**Affiliations:** ^1^Department of Molecular and Cellular Pathology and Therapy, Instituto de Biomedicina de Valencia-CSIC, Valencia, Spain; ^2^Department of Biotechnology, Instituto de Biología Molecular y Celular de Plantas, Universitat Politècnica de València, Valencia, Spain

**Keywords:** mitochondria, respiration, mitophagy, electron transport chain, mitochondrial dysfunction, budding yeast, Atg11

## Abstract

Repair and removal of damaged mitochondria is a key process for eukaryotic cell homeostasis. Here we investigate in the yeast model how different protein complexes of the mitochondrial electron transport chain are subject to specific degradation upon high respiration load and organelle damage. We find that the turnover of subunits of the electron transport complex I equivalent and complex III is preferentially stimulated upon high respiration rates. Particular mitochondrial proteases, but not mitophagy, are involved in this activated degradation. Further mitochondrial damage by valinomycin treatment of yeast cells triggers the mitophagic removal of the same respiratory complexes. This selective protein degradation depends on the mitochondrial fusion and fission apparatus and the autophagy adaptor protein Atg11, but not on the mitochondrial mitophagy receptor Atg32. Loss of autophagosomal protein function leads to valinomycin sensitivity and an overproduction of reactive oxygen species upon mitochondrial damage. A specific event in this selective turnover of electron transport chain complexes seems to be the association of Atg11 with the mitochondrial network, which can be achieved by overexpression of the Atg11 protein even in the absence of Atg32. Furthermore, the interaction of various Atg11 molecules via the C-terminal coil domain is specifically and rapidly stimulated upon mitochondrial damage and could therefore be an early trigger of selective mitophagy in response to the organelles dysfunction. Our work indicates that autophagic quality control upon mitochondrial damage operates in a selective manner.

## Introduction

Mitochondria are membrane-enclosed organelles of eukaryotic cells, which have fundamental functions in energy metabolism via ATP generation by oxidative phosphorylation, in the biogenesis of Fe/S clusters, in the homeostasis of Ca^2+^ and in the regulation of cell death. Mitochondria are considered as the main source of reactive oxygen species (ROS), which can damage a wide range of intracellular components such as lipids, proteins and DNA. Mitochondrial dysfunction is intimately linked to aging and a variety of human diseases, such as neurodegeneration, diabetes and cancer (Wallace, 2005).

Evolutionarily conserved internal mitochondrial proteases seem to act as a first layer of quality control within the organelle. Many of these proteases are ubiquitous in eukaryotic cells and cooperate in the complete degradation of superfluous or damaged mitochondrial proteins (Koppen and Langer, 2007). Failures in the internal mitochondrial proteolysis have severe consequences for cell survival and can induce apoptotic programs and neurodegeneration in mammalian cells. Specifically, ATP dependent AAA proteases located at the inner mitochondrial membrane are important tools of the organelle to remove oxidatively damaged components from the inner membrane (Smakowska et al., 2014).

The selective removal of mitochondria by an autophagy-related process termed mitophagy is essential upon different stimuli, such as starvation or mitochondrial damage. Studies in yeast have revealed many components of the core machinery for autophagy and the biogenesis of the autophagosome (*ATG* genes, Reggiori and Klionsky, 2013). Only some Atg proteins are specific for selective types of autophagy such as mitophagy.

The study of mitophagy in yeast has been almost exclusively focused at starvation induced mitophagy. Yeast cells readily degrade superfluous mitochondria under conditions of severe nutritional stress such as the deprivation of a N-source or in late stationary phase. This experimental setup has been successfully applied to discover the molecular mechanisms of this type of mitophagy (Kanki and Klionsky, 2010; Kanki et al., 2015; Abeliovich and Dengjel, 2016). Here, the Atg32 mitophagy receptor located at the outer mitochondrial membrane is essential (Kanki et al., 2009; Okamoto et al., 2009). Starvation induces multiple phosphorylation at the cytosolic N-terminus of Atg32, which in turn favors the interaction with the autophagosomal adaptor proteins Atg11 and Atg8 (Aoki et al., 2011). Direct phosphorylation of the Atg32 receptor has been shown for casein kinase 2 (Kanki et al., 2013), although at least two other kinases, the MAP kinases Hog1 and Slt2, presumably by indirect mechanisms, trigger starvation induced mitophagy through Atg32 (Mao et al., 2011). Further packaging into autophagosomal vesicles of the mitochondrial parts to be degraded depends on mitochondrial fission at least in some experimental circumstances (Muller et al., 2015). It has been shown that the organelles fission machinery is directly recruited to the autophagosome via the interaction of the Dnm1 protein with the Atg11 adaptor (Mao et al., 2013). Additionally, contact sites between mitochondria and the endoplasmic reticulum (ER) via the ERMES complex seem to be crucial to initiate the autophagosomal engulfment of superfluous parts of the mitochondrial network, presumably by transfer of lipids from the ER to the growing autophagophore (Bockler and Westermann, 2014). However, it is unknown whether mitochondrial damage, rather than nutritional shortage, also induces mitophagic quality control in yeast and whether damage-induced mitophagy implies the same molecular mechanisms of adaptation as compared to starvation (Kanki et al., 2015).

In mammalian cells, it has been well established that damage caused by the depolarization of mitochondria triggers the mitophagic clearance of dysfunctional organelles. Here, PINK1 (PTEN-induced putative kinase 1) and the E3 ubiquitin ligase Parkin are of central importance, and mutations in this system cause the neurodegenerative Parkinson’s disease (Kitada et al., 1998; Valente et al., 2004). PINK1 is localized at the inner mitochondrial membrane in healthy organelles, but gets exposed at the outer mitochondrial membrane upon depolarization (Matsuda et al., 2010; Narendra et al., 2010). As a consequence, Parkin accumulates at the surface of dysfunctional mitochondria in a PINK1 dependent manner, where it ubiquitinates many mitochondrial proteins at the outer membrane (Kazlauskaite and Muqit, 2015). Autophagy receptors such as NDP52 or optineurin are then recruited to the ubiquitinated parts of the mitochondria (Wong and Holzbaur, 2014; Lazarou et al., 2015). Finally, the additional tethering to general autophagy factors such as ULK1, DFCP1 or WIPI1 initiates the local induction of autophagosomal degradation of the damaged mitochondrial portions (Lazarou et al., 2015). The PINK1/Parkin system provides a mechanism of specifically marking damaged mitochondria for their autophagosomal degradation. However, other more general mechanisms exist in mammalian cells for the efficient clearance of superfluous mitochondria in response to developmental and environmental signals. These mechanisms involve outer mitochondrial mitophagy receptors such as Nix or FUNDC1, which are essential for mitophagic pathways induced during erythroid cell maturation (Sandoval et al., 2008; Novak et al., 2010) or by hypoxia (Liu et al., 2012; Wu et al., 2014), respectively.

In more recent studies, an alternative mode of eliminating mitochondrial portions has been described in mammalian cells. Here, the so called mitochondria-derived vesicles (MDV) are responsible for the elimination of oxidized mitochondrial proteins in lysosomes (Soubannier et al., 2012a,b). MDV formation is stimulated by oxidative stress caused by respiratory inhibitors and depends on high respiration rates. Since this mitochondria quality control system does not rely on mitochondrial fission or on the core autophagic machinery, it has been considered an early maintenance system which operates upon milder stress conditions independently on mitophagy (McLelland et al., 2014; Sugiura et al., 2014).

Leakage of electrons from the mitochondrial electron transport chain (ETC) has been confirmed to account for most of intracellar reactive oxygen species (ROS) in eukaryotic organisms (Murphy, 2009). Therefore an efficient quality control of the different ETC complexes is important for cell homeostasis and cell survival. Almost 50 years ago, it was suggested that a strict quality control of individual ETC multiprotein complexes was based on the functionality of each subcomplex (Luzikov et al., 1970). Over the past decades, many studies have built up on this concept and confirmed that mitochondrial multisubunit complexes are subject to multiple proteolytic control, either inside the organelle or by vacuolar/lysosomal mechanisms (Luzikov, 2002, 2009). In this regard, it is important to note that mitochondrial protein turnover is not restricted to starvation or internal or external damage conditions. The use of synchronous yeast cultures has shown that mitochondrial respiratory performance suffers oscillatory changes in proliferating cells (Bashford et al., 1980; Lloyd et al., 2002). Thus, the turnover of specific mitochondrial components such as cytochromes or ATP synthase are dynamically adjusted in metabolically active cells (Lloyd, 2008).

It is largely unknown whether mitophagy is a general mechanism for the bulk elimination of excessive or malfunctioning mitochondria or whether it can more specifically remove selected portions from the organelles network. Until now it has been even questioned whether specific mitochondrial damage can induce mitophagic repair mechanisms in yeast. Here we address these questions by following the fate of individual subunits of the different mitochondrial electron transport complexes (ETC) upon the activation of respiratory metabolism or valinomycin triggered damage. We find large differences in the turnover of individual ETCs, with CI and CIII suffering a selective degradation upon high respiration rates. Protein subunits of the same complexes are preferentially degraded upon valinomycin-induced mitochondrial damage, which depends on the selective autophagy adaptor Atg11, but not on the mitophagy receptor Atg32. Furthermore, the damage-induced dimerization or oligomerization of Atg11 might be an early trigger in this selective mitophagy.

## Materials and Methods

### Yeast Strains and Growth Conditions

*Saccharomyces cerevisiae* strains used in this study are shown in **Table 1**. Yeast strains were grown at 28°C yeast extract-peptone containing 2% dextrose (YPD), 2% galactose (YPGal) or 3% glycerol (YPGlyc) with or without the indicated supplementation of valinomycin. Synthetic dextrose (SD) or galactose (SGal) media contained 0.67% yeast nitrogen base, 50 mM succinic acid (pH 5.5) and 2% of the respective energy source. According to the auxotrophies of each strain, methionine (10 mg/l), histidine (10 mg/l), leucine (10 mg/l), or uracil (25 mg/l) were added. For the estimation of growth efficiencies, fresh overnight precultures were diluted in triplicate in multiwell plates to the same OD. Growth was then continuously monitored under the indicated conditions in a Bioscreen C system (Thermo) for the indicated times. For the degradation assays for specific respiratory complex subunits, cells expressing the indicated protein as a TAP fusion from the genomic locus were pregrown in SGal medium to exponential phase and then treated for the indicated times with 4 μM valinomycin.

**Table 1:** Yeast strains used in this study.

Name	Relevant genotype	Source
BY4741	*MATa*; *his3Δ1*; *leu2Δ0*; *met15Δ0*; *ura3Δ0*	EUROSCARF
Nde1-TAP	BY4741 with *NDE1-TAP::HIS3MX6*	Ghaemmaghami et al., 2003
Ndi1-TAP	BY4741 with *NDI1-TAP::HIS3MX6*	Ghaemmaghami et al., 2003
Sdh2-TAP	BY4741 with *SDH2-TAP::HIS3MX6*	Ghaemmaghami et al., 2003
Qcr2-TAP	BY4741 with *QCR2-TAP::HIS3MX6*	Ghaemmaghami et al., 2003
Cox6-TAP	BY4741 with *COX6-TAP::HIS3MX6*	Ghaemmaghami et al., 2003
Atp5-TAP	BY4741 with *ATP5-TAP::HIS3MX6*	Ghaemmaghami et al., 2003
Cyc1-TAP	BY4741 with *CYC1-TAP::HIS3MX6*	Ghaemmaghami et al., 2003
Fba1-TAP	BY4741 with *FBA1-TAP::HIS3MX6*	Ghaemmaghami et al., 2003
Qcr2-TAP atg11	BY4741 with *QCR2-TAP::HIS3MX6 atg11::KanMX*	This study
Qcr2-TAP atg32	BY4741 with *QCR2-TAP::HIS3MX6 atg32::KanMX*	This study
Qcr2-TAP yme1	BY4741 with *QCR2-TAP::HIS3MX6 yme1::KanMX*	This study
Qcr2-TAP pim1	BY4741 with *QCR2-TAP::HIS3MX6 pim1::KanMX*	This study
Qcr2-TAP afg3	BY4741 with *QCR2-TAP::HIS3MX6 afg3::KanMX*	This study
Qcr2-TAP fzo1	BY4741 with *QCR2-TAP::HIS3MX6 fzo1::KanMX*	This study
Qcr2-TAP fis1	BY4741 with *QCR2-TAP::HIS3MX6 fis1::KanMX*	This study
Ndi1-TAP atg11	BY4741 with *NDI1-TAP::HIS3MX6 atg11::KanMX*	This study
Ndi1-TAP atg32	BY4741 with *NDI1-TAP::HIS3MX6 atg32::KanMX*	This study
Ndi1-TAP fzo1	BY4741 with *NDI1-TAP::HIS3MX6 fzo1::KanMX*	This study
Ndi1-TAP fis1	BY4741 with *NDI1-TAP::HIS3MX6 fis1::KanMX*	This study
BY4741 mtGFP	BY4741 with plasmid pVT100U-mtGFP (*URA3*; *pADH1-mtGFP*)	Westermann and Neupert, 2000
Atp5-GFP	BY4741 with *ATP5-GFP::HIS3MX6*	Huh et al., 2003
Ndi1-GFP	BY4741 with *NDI1-GFP::HIS3MX6*	Huh et al., 2003
Qcr2-GFP	BY4741 with *QCR2-GFP::HIS3MX6*	Huh et al., 2003
BY4741 mtRosella	BY4741 with plasmid pVT100U-mtRosella (*URA3*; *pADH1-mtRosella*)	Bockler and Westermann, 2014
atg11 mtRosella	BY4741 *atg11::KanMX* with plasmid pVT100U-mtRosella (*URA3*; *pADH1-mtRosella*)	This study
atg32 mtRosella	BY4741 *atg32::KanMX* with plasmid pVT100U-mtRosella (*URA3*; *pADH1-mtRosella*)	This study
BY4741 Atg11-dsRed	BY4741 with plasmid pAG415-GPD-ATG11-DsRed (*LEU2*; *CEN*; *pTDH3-ATG11-dsRed*)	This study
atg32 Atg11-dsRed	BY4741 *atg32::KanMX* with plasmid pAG415-GPD-ATG11-DsRed (*LEU2*; *CEN*; *pTDH3-ATG11-dsRed*)	This study
Qcr2-TAP Atg11-dsRed	BY4741 *QCR2-TAP::HIS3MX6* with plasmid pAG415-GPD-ATG11-DsRed (*LEU2*; *CEN*; *pTDH3-ATG11-dsRed*)	This study
Ndi1-TAP Atg11-dsRed	BY4741 *NDI1-TAP::HIS3MX6* with plasmid pAG415-GPD-ATG11-DsRed (*LEU2*; *CEN*; *pTDH3-ATG11-dsRed*)	This study
BY4741 mtRosella Atg11-dsRed	BY4741 with plasmids pAG415-GPD-ATG11-DsRed (*LEU2*; *CEN*; *pTDH3-ATG11-dsRed*) and pVT100U-mtRosella (*URA3*; *pADH1-mtRosella*)	This study
atg32 mtRosella Atg11-dsRed	BY4741 *atg32::KanMX* with plasmids pAG415-GPD-ATG11-DsRed (*LEU2*; *CEN*; *pTDH3-ATG11-dsRed*) and pVT100U-mtRosella (*URA3*; *pADH1-mtRosella*)	This study
THY.AP4	*MATa*; *ura3; leu2::lexA; lacZ::trp1; lexA::HIS3; lexA::ADE2*	Paumi et al., 2007
THY.AP4 pACT2, pBTM116	THY.AP4 with empty plasmids pACT2 and pBTM116	This study
THY.AP4 pACT2-ATG11, pBTM116	THY.AP4 with plasmid pACT2-ATG11_(969-1179)_ and empty plasmid pBTM116	This study
THY.AP4 pACT2, pBTM116-ATG11	THY.AP4 with empty plasmid pACT2 and plasmid pBTM116-ATG11	This study
THY.AP4 pACT2-ATG11, pBTM116-ATG11	THY.AP4 with plasmids pACT2-ATG11_(969-1179)_ and pBTM116-ATG11	This study
Atg11-TAP	BY4741 with *ATG11-TAP::HIS3MX6*	Ghaemmaghami et al., 2003
Atg11-TAP	BY4741 with *ATG11-TAP::HIS3MX6* and plasmid	
Atg11-HA	pAG416-GPD-ATG11-HA (*URA3*; *CEN*; *pTDH3-ATG11-HA*)	This study

### Plasmid Constructions

We used plasmid pVT100U-mtGFP, which contains mitochondrially targeted GFP expressed from the *ADH1* promoter, to visualize the complete mitochondrial network in yeast (Westermann and Neupert, 2000). For the microscopic survey of mitophagy we used plasmid pVT100-mtRosella, which contains the dual fluorescent indicator Rosella targeted to mitochondria expressed from the *ADH1* promoter (Mijaljica et al., 2011). For constitutive overexpression of Atg11 fused with dsRed from the *TDH3* promoter, we inserted the entire *ATG11* gene into plasmid pAG415-GPD-dsRed (Alberti et al., 2007). For the constitutive expression of HA-tagged Atg11 expressed from the *TDH3* promoter, we inserted the entire *ATG11* gene into yeast expression plasmid pAG416-GPD-HA (Alberti et al., 2007). A yeast two-hybrid bait plasmid containing lexA-Atg11 expressed from the *ADH1* promoter was constructed by insertion of the *ATG11* gene into plasmid pBTM116.

### Immunological Methods

Yeast whole cell extracts were prepared instantaneously by boiling the cell pellet from 10 ml of fresh culture in 2x Laemmli buffer (120 mM Tris/HCl pH 6.8; 3% SDS; 40 mM DTT; 4 mM EDTA; 12% saccharose; 0.1 mg/ml bromphenolblue) for 5 min. Proteins were separated by 10% SDS-PAGE and analyzed by immunoblotting on PVDF membranes using anti-peroxidase-anti-peroxidase (anti-PAP) antibody (Sigma; 1:10.000) and peroxidase labeled anti-rabbit (Amersham Biosciences; 1:10.000). In the coprecipitation experiments, we additionally applied an anti-HA antibody (12CA5 Roche; 1:10.000). The bands were visualized with ECL Plus (Amersham Biosciences) and quantified with a Fujifilm LAS3000 or ImageQuant LAS4000 system. DB71 staining of the membranes was used as a loading control (Hong et al., 2000).

### Fluorescence Microscopy

Vacuoles were stained with CellTracker Blue CMAC (Life Technologies) in living cells. Cells were observed on a Leica confocal microscope LSM 780 using the following excitation and emission wavelengths: GFP/pHluorin (excitation 488 nm; emission 509 nm), dsRed (excitation 545 nm; emission 572 nm), CMAC (excitation 353 nm; emission 466 nm).

### Measurement of Oxygen Consumption

For the quantification of the respiration rate, yeast cells were exponentially grown in SGal medium, washed with water and finally resuspended in 40 mM NaPO_4_ pH 7.4 with 1% glucose. Oxygen consumption was then quantified in intact cells using a Mitocell S200 Respiratory System (Strathkelvin Instruments) with a Clarke type oxygen electrode. Oxygen consumption rates were determined from at least three independent culture aliquots.

### Two Hybrid Screening

The entire *ATG11* gene was cloned into plasmid pBTM116 to create a full length Atg11-lexA fusion used as the bait in the two hybrid screening. A genomic yeast library cloned in the pACT plasmid fused to the Gal4 transcriptional activation domain was co-transformed with the Atg11-lexA bait vector. Total transformants were collected from SD-trp-leu selective plates in TE buffer and then plated in different dilutions onto SD-trp-leu-his-ade plates containing 4 μM Valinomycin. Positive clones were verified by colorimetric β-gal assays on filter papers and quantitative β-gal assays using liquid yeast cultures.

### Coprecipitation Experiments

Atg11-TAP expressing yeast cells were transformed with the Atg11-HA expression plasmid or the empty vector control. The transformants were grown to exponential phase under the indicated conditions and total protein extracts were obtained by glass bead lysis in buffer A (50 mM Tris HCl pH 7.5; 15 mM EDTA; 2 mM DTT; 150 mM NaCl; 1 mM PMSF; 0.1% Triton X-100). Equal amounts of total protein were incubated with Dynabeads Pan Mouse IgG (Invitrogen) for 2 h in the cold. Extensive washing with buffer A was followed by extract preparation in 2x Laemmli buffer and heating for 5 min at 95°C. Precipitated TAP proteins were analyzed by anti-PAP western blot, while coprecipitated HA-tagged proteins were analyzed by anti-HA western blot.

### ROS Quantification

Exponential cell culture aliquots upon the indicated growth conditions were incubated for 30 min with 2′, 7′-dichlorodihydrofluorescein diacetate (H_2_DCFDA; Sigma) at a final concentration of 10 μM. H_2_DCFDA is a chemically reduced form of fluorescein and used as a quantitative indicator of ROS levels in living cells. Inside the cell, it is cleaved by intracellular esterases, while further oxidation converts it to the fluorescent 2′, 7′-dichlorofluorescein. The cells were washed with water and resuspended in 1 ml of 50 mM Tris/HCl pH 7.5. After the addition of 10 μl of chloroform and 5 μl of 0.1% SDS, cell extracts were prepared by rigorous agitation (max speed for 30 s in a vortex homogenizor) with glass beads (0.5 mm diameter). Fluorescence was quantified in the supernatant in a microplate reader at 492 nm excitation and 525 nm emission wavelengths and normalized for the fluorescence of the same number of mock-treated cells.

### Cycloheximide Chase Experiments

Cycloheximide chase experiments were performed using yeast strains expressing different TAP-tagged subunits of the mitochondrial electron transport chain from their genomic loci. The experimental procedure is described in (Buchanan et al., 2016). Cycloheximide (Sigma) was used at a final concentration of 250 μg/ml in exponentially grown yeast culture aliquots.

## Results

### Electron Transport Chain Complexes I and III Are Preferentially Degraded upon High Respiration Rates

**FIGURE 1:**
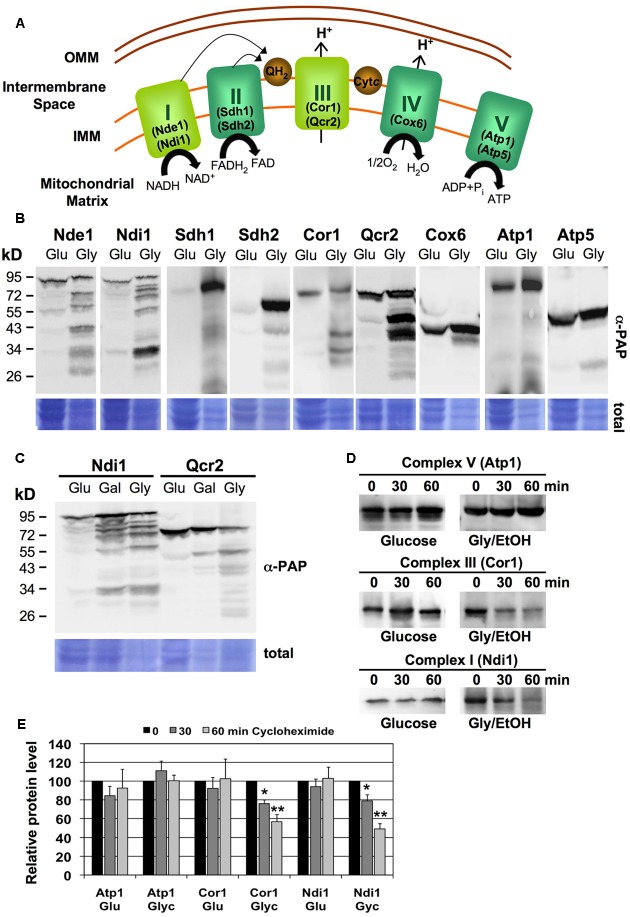
Turnover of specific mitochondrial respiratory complexes upon high respiration rates. **(A)** Schematic overview of the yeast mitochondrial electron transport chain. The specific subunits used in this work are depicted. I,NADH:ubiquinone oxidoreductase; II, Succinate dehydrogenase; III, Cytochrome bc1 complex; IV, Cytochrome c oxidase; V, ATP synthase. Please note that NADH:ubiquinone oxidoreductase (CI equivalent) exists in budding yeast as the single polypeptides Ndi1 or Nde1, while all other ETC units are multisubunit complexes. **(B)** Subunits of CI and CIII undergo massive degradation upon respiratory growth. Whole cell extracts of yeast strains expressing the indicated subunits as TAP fusion proteins were analyzed by western blot upon glucose (Glu) or glycerol (Gly) growth. **(C)** Ndi1 and Qcr2 protein degradation was assessed by western blot as described above upon glucose (Glu), galactose (Gal) or glycerol (Gly) growth. **(D)** Yeast strains expressing the Atp1, Ndi1 or Cor1 subunits as TAP fusion proteins on fermentative medium (Glucose) or respiratory medium (Glycerol/EtOH) were treated for the indicated times with cycloheximide. Full length proteins were then visualized by anti-PAP western blot. Representative blots are shown for each strain and condition. **(E)** Quantification of the blots described in **(D)**. Three independent experiments were analyzed. Data shown are mean values with the standard deviation. Significantly different protein levels with respect to the untreated control are marked: ^∗^*p* < 0.05; ^∗∗^*p* < 0.01 (Student’s *t*-test).

We studied the abundance of individual subunits of the five respiratory complexes in yeast upon low respiratory rates during glucose growth or upon highly induced respiration during glycerol growth (**Figure 1**). We employed fully functional TAP-tagged versions of the different ETC subunits: Nde1 and Ndi1 (CI), Sdh1 and Sdh2 (CII), Cor1 and Qcr2 (CIII), Cox6 (CIV), and Atp1 and Atp5 (CV) (**Figure 1A**). Although budding yeast has single alternative oxidases (NADH:ubiquinone oxidoreductases Ndi1 and Nde1) replacing the function of the multisubunit ETC complex I present in higher eukaryotes, they are named as CI throughout the work to facilitate the presentation of the results. All proteins tested were detected in whole cell extracts as a major band corresponding to the full-length protein upon fermentative glucose growth. However, in cells actively growing in non-fermentative media, we observed the appearance of many degradation products in the case of the Nde1, Ndi1, Cor1 and Qcr2 proteins representing mitochondrial complexes CI and CIII (**Figure 1B**). In all cases, the abundance of multiple degradation bands was actually greater than the full length protein, which indicated that active respiration in yeast cells was accompanied by an important turnover of CI and CIII respiratory complexes.

We next addressed the question whether the increased degradation of certain respiratory complexes correlated with the respiration rate of the cells. The distribution of full length and degradation products of the Ndi1 and Qcr2 proteins was assayed in yeast cells grown in fully fermentative (glucose), partially respiratory (galactose) or fully respiratory (glycerol) conditions. As shown in **Figure 1C**, the percentage of degradation of the two proteins increased gradually with the increased dependence of the cells on respiratory metabolism.

Protein steady state levels are determined by the rates of their degradation and de novo synthesis. In order to determine the half-life of specific ETC subunits, we performed cycloheximide chase experiments upon fermentative and respiratory growth conditions. We found that the CV subunit Atp1 was stable during 1 h of impaired protein synthesis independently of the respiration rate (**Figure 1D**). The same result was obtained for the CI and CIII subunits Ndi1 and Cor1 upon fermentative conditions, which, however, were rapidly degraded upon respiratory growth (**Figures 1D,E**). These results suggested that specific ETC complexes, namely CI and CIII, are more rapidly degraded in yeast cells, which actively respire.

**FIGURE 2:**
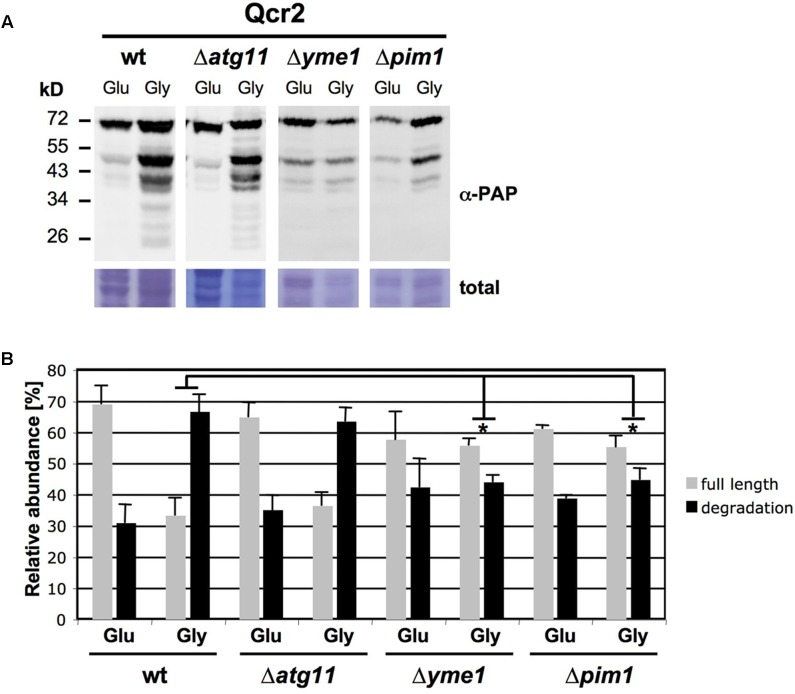
Enhanced degradation of Qcr2 upon respiratory growth depends on the mitochondrial proteases Yme1 and Pim1. **(A)** Qcr2 protein degradation in the indicated mutant strains was assessed by western blot as described in **Figure 1**. A representative blot is shown. **(B)** Quantification of the ratio of full length and degradation products of the Qcr2 protein. Mean values with the standard deviation are shown based on three independent experiments. Significantly different degradation rates between different strains are marked: ^∗^*p* < 0.05 (Student’s *t*-test). All other comparisons were not statistically different.

We next wanted to gain insights into the mechanisms underlying the respiration rate dependent degradation of mitochondrial complexes. We focused at the CIII subunit Qcr2 and analyzed its induced degradation in mitophagy mutants (*atg11*, *atg32*) and mutants with a lack of function of specific internal mitochondrial proteases (*afg3*, *pim1*, *yme1*). As shown in **Figure 2**, only mutations of the inner mitochondrial membrane i-AAA protease Yme1 and the Lon mitochondrial matrix protease Pim1 significantly decreased the percentage of Qcr2 degradation upon respiratory growth. These results indicated that the enhanced degradative turnover of respiratory complexes upon active respiration depends on the activity of specific mitochondrial proteases and not on mitophagy.

### Valinomycin-Triggered Damage Induces the Degradation of ETCI and III via a Mitophagic Mechanism Independent of Atg32

**FIGURE 3:**
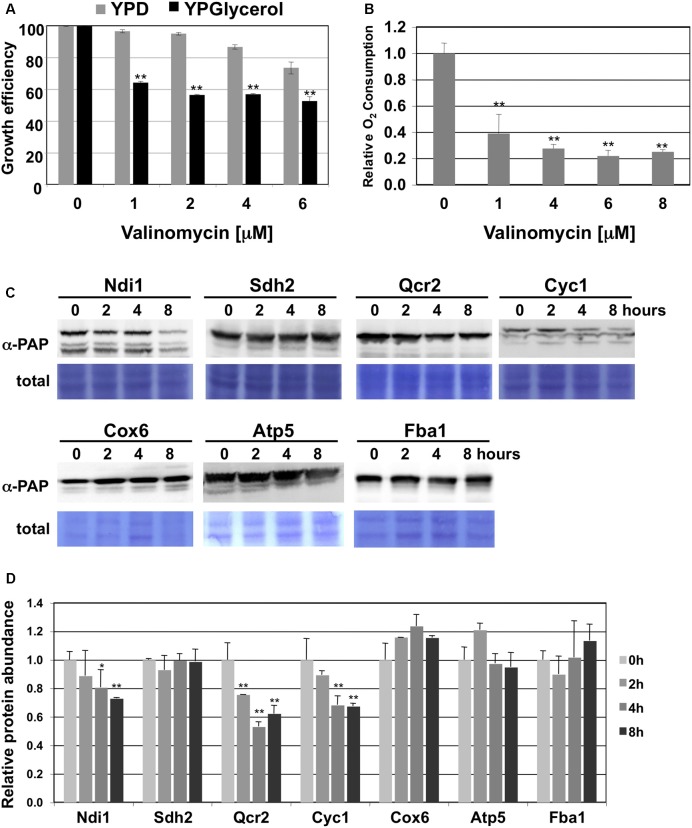
CI and CIII subunits of the mitochondrial electron transport chain are preferentially degraded upon valinomycin stress. **(A)** Comparison of the growth inhibition of yeast wild type cells by valinomycin upon fermentative (YPD) or respiratory (YPGlycerol) conditions. The growth of three independent cultures was continuously analyzed for each condition. Mean values are depicted including the standard deviation. ^∗∗^*p* < 0.01 (Student’s *t*-test). **(B)** Effect of valinomycin treatment on the O_2_ consumption rates of yeast wild type cells. Cells were grown in galactose containing medium and then treated with the indicated doses of valinomycin. Data are mean values from three independent measurements with the standard deviation. ^∗∗^*p* < 0.01 (Student’s *t*-test). **(C)** Subunits of CI and CIII are selectively degraded upon valinomycin treatment. Whole cell extracts of yeast strains expressing the indicated subunits as TAP fusion proteins were analyzed by western blot during the treatment with 4 μM valinomycin in galactose containing media. Ndi1 (CI), Sdh2 (CII), Qcr2 (CIII), Cyc1 (CIII), Cox6 (CIV) and Atp5 (CV) were used as representatives of the mitochondrial electron transport complexes. Fba1 served as a glycolytic enzyme control. α-PAP indicates the detection of TAP fusion proteins. Representative blots are shown from a total of three independent experiments. **(D)** Quantification of the protein abundance upon the conditions described above. The standard error is shown based on three independent experiments. Significantly different degradation rates are marked: ^∗^*p* < 0.05; ^∗∗^*p* < 0.01 (Student’s *t*-test).

The accelerated degradation of CI and CIII subunits of the mitochondrial electron transport chain could be the result of the accumulation of damage at these specific complexes upon heavy respiration. The proteolytic removal of CI and CIII could therefore be necessary to maintain the overall function of the mitochondrial electron transport chain. We wanted to go a step further and test how the turnover of specific ETCs responds to mitochondrial damage. We chose the antibiotic valinomycin to provoke mitochondrial dysfunction, because it locally disrupts the mitochondrial membrane potential independently of a specific ETC by the formation of K^+^ selective pores. We first confirmed the suitability of valinomycin treatment of yeast cells as a model to study the adaptation to mitochondrial damage. As shown in **Figure 3**, valinomycin inhibits yeast growth in the low μM range specifically upon respiratory conditions and reduces the O_2_ consumption rate up to 80%. We next performed kinetic quantitative western analysis to measure the effect of valinomycin treatment on the turnover of subunits of the individual ETCs. We found an increased degradation over time specifically at the CI and CIII subunits Ndi1 and Qcr2 and for cytochrome c, but not at CII, CIV or CV (**Figures 3C,D**). These results indicated that also externally induced mitochondrial dysfunction induces ETC specific protein degradation in yeast.

**FIGURE 4:**
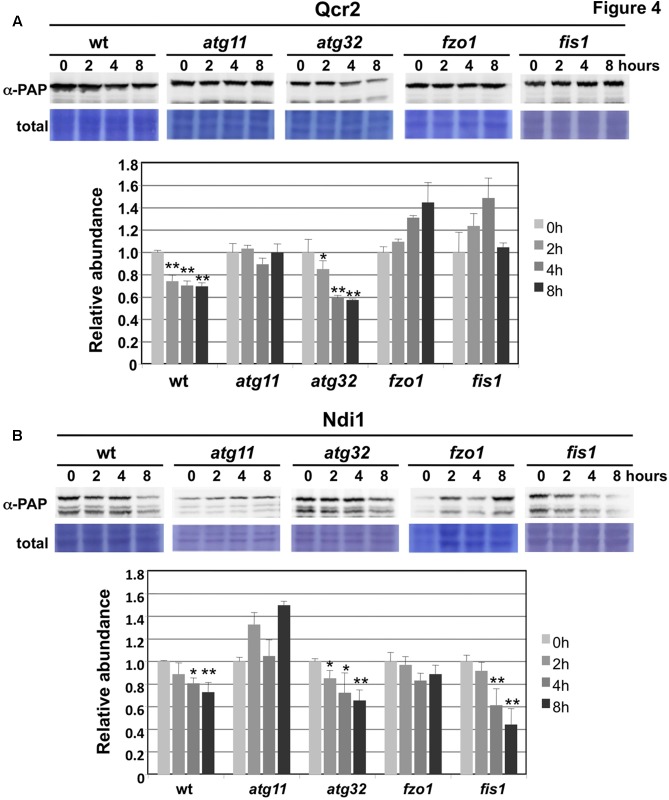
Degradation of CI and CIII subunits upon mitochondrial damage is dependent on Atg11 and mitochondrial fusion/fission, but independent on Atg32. Experimental conditions were the same as in **Figure 3**. **(A)** CIII subunit Qcr2 degradation upon valinomycin treatment in wild type and the indicated deletion mutants. Data are shown as mean values from three independent experiments including the standard error. **(B)** Ndi1 degradation upon valinomycin treatment in wild type and the indicated deletion mutants. Data are shown as mean values from three independent experiments including the standard error. Significantly different degradation rates are marked: ^∗^*p* < 0.05; ^∗∗^*p* < 0.01 (Student’s *t*-test).

We next tested whether mitophagy or the function of the mitochondrial fusion and fission machinery was implied in the observed selective degradation of mitochondrial proteins upon valinomycin treatment. We quantified the abundance of the Ndi1 and Qcr2 proteins during valinomycin exposure in the mitophagy mutants *atg11* and *atg32* and in the fusion/fission mutants *fzo1* and *fis1* (**Figures 4A,B**). We found that the mitophagy adaptor Atg11 was essential to decrease Ndi1 and Qcr2 protein levels upon damage. However, the Atg32 mitophagy receptor was dispensable in this process. Furthermore, we confirmed that mitochondrial dynamics, both fusion and fission, were important for efficient Qcr2 degradation, while for Ndi1 degradation we only observed a dependence on mitochondrial fusion. These data suggested that valinomycin-induced mitochondrial damage triggered a mitophagic response dependent on mitochondrial dynamics to degrade specific ETCs from the organelles network. However, specific respiratory complexes might depend on mitochondrial fusion and fission processes to a different degree.

**FIGURE 5:**
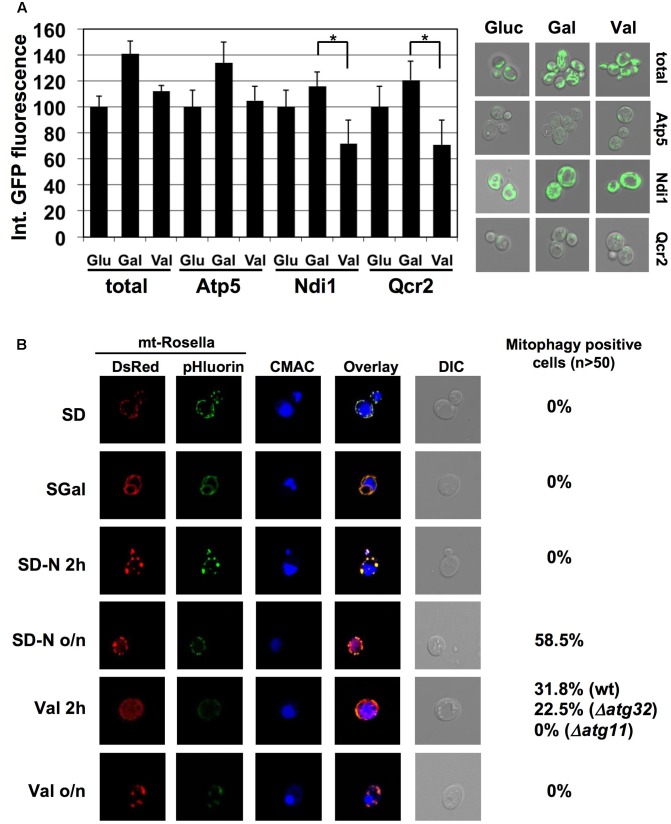
Valinomycin damage induces ETC specific mitophagy in yeast. **(A)** GFP fluorescence from the indicated ETC subunits or from mt-GFP was quantified upon fermentative growth (Glu) or partially respiratory growth without (Gal) or after treatment for 2 h with 4 μM valinomycin (Val). The mean of the integrated fluorescence intensity acquired from flattened z-stack images for >50 cells was calculated in one experiment. Data are shown as mean values and the standard error from two independent experiments. Asterisks mark a significant signal reduction (*p* < 0.05) according to the Students *t*-test. Representative micrographs are depicted at the right. **(B)** Valinomycin rapidly induces mitophagy in yeast cells. The mitophagy indicator Rosella was used in the indicated yeast strains. Cells were grown in selective glucose containing (SD) or galactose containing (SGal) media. Galactose grown cells were additionally subjected to N-starvation or valinomycin treatment (4 μM) for 2 h or over night as indicated. mtRosella was visualized by dsRed or pHluorin fluorescence, vacuoles were visualized with CMAC. The percentage of mitophagy positive cells is indicated at the right.

We wanted to monitor a selective removal of specific ETC subunits from yeast mitochondria upon damage by alternative approaches. We therefore quantified the abundance of functional GFP-fusion proteins of CI (Ndi1), CIII (Qcr2) and CV (Atp5) by confocal fluorescence microscopy and compared it to whole mitochondria visualized by mt-GFP. We acquired the integrated GFP intensities in each case over a cell population upon fermentative growth (glucose), partially respiratory growth (galactose) and after valinomycin treatment (**Figure 5A**). Expectedly, all mitochondrial ETC proteins increased during the shift from glucose to galactose growth similarly to the observed extension of the total mitochondrial network. Treatment of galactose grown cells with valinomycin reduced the total mitochondrial signal approximately to the level of glucose repressed cells, and a similar decrease was observed for the CV subunit Atp5. However, Ndi1- and Qcr2-GFP levels decreased significantly more upon external mitochondrial damage. These data indicated again that CI and CIII subunits are removed from the mitochondrial network upon damage in a selective manner.

In order to confirm our genetic data which indicated that a selective and Atg32 independent mitophagy occurred after mitochondrial damage in yeast, we employed the Rosella indicator to visualize the extent of autophagic digestion of mitochondrial parts in the vacuole upon valinomycin-induced damage. Rosella is directed to mitochondria by a specific targeting sequence and contains a pH-sensitive GFP fused to red fluorescent protein (dsRed). Upon normal growth, red and green light emission overlaps completely in mitochondria, whereas during mitophagy mtRosella enters the vacuole where the green signal is lost due to the acidic pH. As shown in **Figure 5B**, glucose or galactose cultures did not contain mitophagy-positive cells. However, treatment of galactose grown cells with valinomycin rapidly induced the percentage of cells, which engaged in mitophagy. Interestingly, at this short time point, the deprivation of a N-source, which is the standard treatment to induce autophagic degradation of mitochondria in yeast cells, did not produce mitophagy positive cells. However, vacuolar degradation of mitochondria was observed after prolonged N-starvation (**Figure 5B**). Taken together, we confirmed that valinomycin induced damage leads to the rapid removal of mitochondrial parts in the vacuole in a more instantaneous manner as compared to starvation conditions. We next tested the implication of the known mitophagy mediators Atg11 and Atg32. While Atg11 was absolutely necessary to induce mitochondrial degradation at the vacuole upon valinomycin damage, in *atg32* mutant cells an induction of mitophagy-positive cells was still observed, which was only slightly reduced as compared to wild type (**Figure 5B**).

**FIGURE 6:**
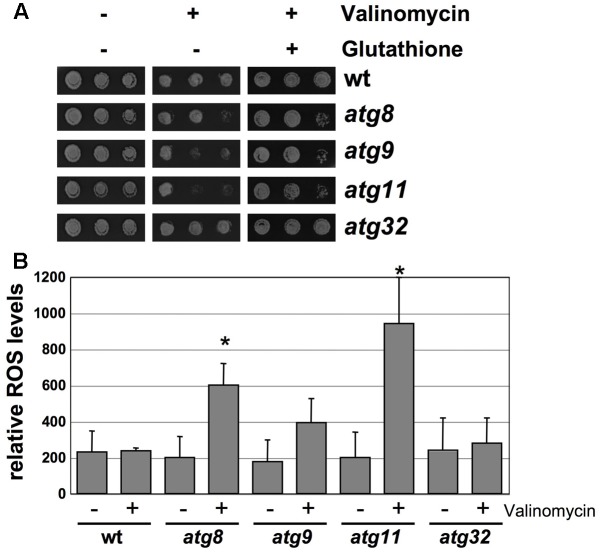
Loss of autophagosomal functions impairs adaptation to valinomycin. **(A)** The indicated yeast strains were grown in YPGal medium and treated or not with 4 μM valinomycin in the presence or absence of 5 mM glutathione for 12 h in serial dilutions. Colony formation was then assessed on YPD agar plates. **(B)** Reactive oxygen species (ROS) in the same yeast strains. The 2′7′-dichlorodihydrofluorescein diacetate assay was performed before and after treatment with valinomycin (4 μM, 2 h). Data represent the mean values from three independent assays. Significantly increased ROS levels relative to wt are marked. ^∗^*p* < 0.05 (Student’s *t*-test).

We next addressed the physiological relevance of the mitochondrial protein turnover by mitophagy upon valinomycin damage described above. The K^+^-ionophore valinomycin is known to induce an overproduction of mitochondrial ROS (Andrukhiv et al., 2006; Selivanov et al., 2008). As shown in **Figure 6A**, loss of function of the autophagic core proteins Atg8, Atg9 or Atg11 rendered yeast cells hypersensitive to valinomycin stress. By contrast, deletion of Atg32 did not produce a detectable phenotype. The treatment with the antioxidant glutathione partially rescued the sensitivity of the *atg8*, *9* and *11* mutants. Additionally we quantified the intracellular ROS accumulation before and after valinomycin treatment in wild type and the same autophagy mutants. As depicted in **Figure 6B**, the *atg8*, *atg9,* and *atg11* mutants showed increased ROS levels upon mitochondrial damage, which was statistically significant in the *atg8* and *atg11* strains. Taken together, our results indicated that the core autophagic machinery including the Atg11 adaptor, but not Atg32, played an essential role in the damage-induced selective mitophagy, which is a physiologically important function for the removal of dysfunctional parts of the organelle.

### Atg11 Homodimerization and Association with the Mitochondrial Network Are Essential Steps in Valinomycin Induced Selective Mitophagy

**FIGURE 7:**
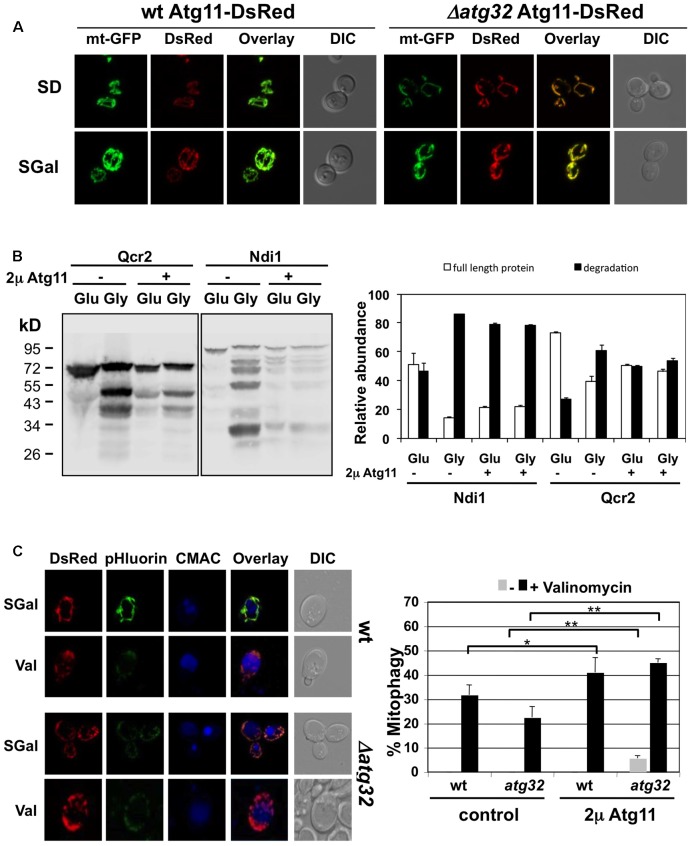
Effects of constitutive Atg11 targeting to mitochondria. **(A)** Constitutive overexpression leads to Atg11 association with mitochondria. Atg11-dsRed was overexpressed in yeast wild type or *atg32* mutant cells harboring mtGFP. The mitochondrial network was visualized by GFP fluorescence, while Atg11 was visualized by dsRed upon fermentative (SD) and partially respiratory (SGal) growth conditions. **(B)** Qcr2 and Ndi1 protein degradation was assessed by western blot using chromosomally expressed TAP-fusions. Cells overexpressed the Atg11-dsRed fusion protein or contained the empty vector as indicated. Quantification of the ratio of full length and degradation products of the Qcr2 and Ndi1 proteins is shown in the right panel. The standard error is shown based on three independent experiments. **(C)** Constitutive Atg11 expression reverts mitophagy defects of *atg32* mutants, but does not lead to constitutive mitophagy induction. The mitophagy indicator Rosella was used in yeast wild type and *atg32* mutant strains in the presence or absence of Atg11 overexpression. Cells were grown in selective galactose containing (SGal) media and then treated with valinomycin (4 μM) for 2 h. Representative micrographs are shown for the Atg11 overexpression strains at the left. mtRosella was visualized by dsRed or pHluorin fluorescence, vacuoles were visualized with CMAC. The percentage of mitophagy positive cells was determined from >50 individual cells in two independent experiments. The mean values including the standard error are indicated in the right panel. Significant changes in the rate of mitophagy positive cells are marked: ^∗^*p* < 0.05; ^∗∗^*p* < 0.01 (Student’s *t*-test).

In order to reveal mechanistic insights into the selective removal of damaged mitochondria in yeast, we studied the function of the Atg11 adaptor. Atg11 is present in yeast cells at a very low copy number (Ghaemmaghami et al., 2003) and we have confirmed that Atg11-GFP expressed at endogenous levels is practically undetectable in normally growing cells. We therefore studied the effects of a gain of Atg11 function and constitutively overexpressed the protein as a fusion with dsRed. Unexpectedly we found that overexpressed Atg11 localizes to the mitochondria, as shown in **Figure 7A**. Moreover, mitochondrial targeting of Atg11 was independent of Atg32, a known interactor for Atg11 at the mitochondrial outer membrane upon starvation. We then asked whether overexpressed Atg11 at mitochondria provoked changes in the turnover of CI and CIII subunits of the electron transport chain. We measured the percentage of degradation, which occurred at the Ndi1 and Qcr2 proteins upon the shift from fermentation to respiration in the absence and presence of Atg11 overexpression. As shown in **Figure 7B**, both subunits suffered an enhanced degradation already upon fermentative growth in the Atg11 gain of function strain. This degradation rate was comparable to the rate observed upon full induction of respiration. We next analyzed the valinomycin-induced mitophagy in the context of Atg11 overexpression with the Rosella indicator. Mitophagy-positive cells were exclusively detected after the induction of mitochondrial damage even during the constitutive overexpression of Atg11 (**Figure 7C**). Of note, Atg11 overexpression increased the number of cells committed to mitophagy slightly in the wild type and notably in the *atg32* mutant. These date indicated that Atg11 association with mitochondria is an important step in the induction of mitophagy. However, mitochondrially targeted Atg11 alone is not sufficient to trigger mitophagy, which remains completely dependent on mitochondrial damage.

**FIGURE 8:**
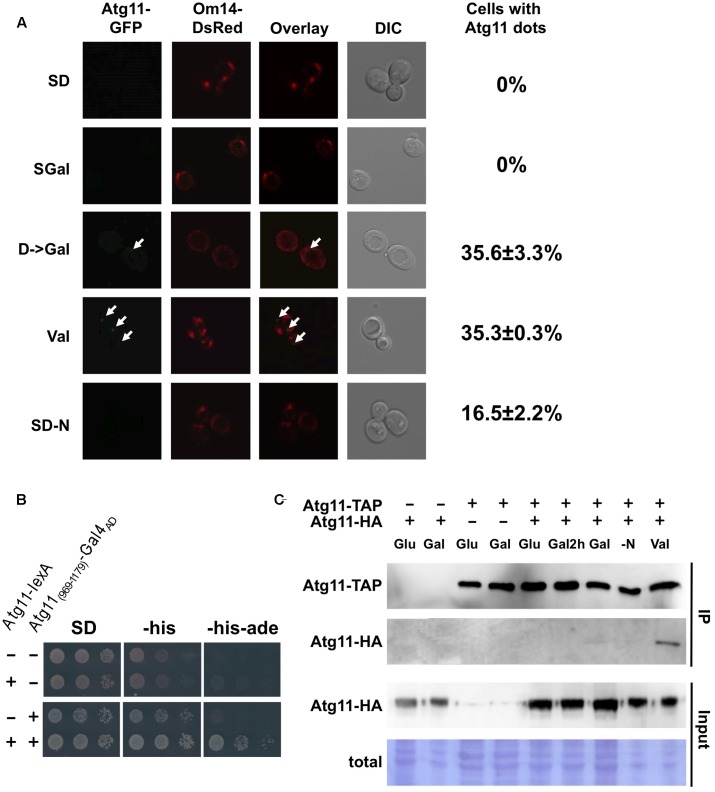
Valinomycin-mediated damage induces Atg11 oligomerization and Atg11 containing autophagosomal bodies near mitochondria. **(A)** Endogenously expressed Atg11-GFP was visualized in yeast cells grown in glucose (*SD*), galactose (SGal), shifted for 2 h from glucose to galactose, treated for 2 h with valinomycin (4 μM), or depleted for nitrogen for 2 h. The outer mitochondrial membrane protein Om14 fused to dsRed was used to stain the entire mitochondrial network. The appearance of Atg11 dots is indicated with arrows in the micrographs. The percentage of cells with Atg11 autophagosomal bodies is given at the right and was determined from > 50 individual cells in two independent experiments. The mean values including the standard error are indicated. **(B)** The C-terminal (969–1179) domain of Atg11 is sufficient for homo dimerization. Full length Atg11 expressed as a lexA fusion from plasmid pBTM116 and/or Gal4_AD_-Atg11_(969-1179)_ expressed from plasmid pACT2 was used as indicated in yeast THY.AP4 strain. Two hybrid interaction was confirmed by growth in media lacking histidine and adenine. **(C)** Atg11 dimerization is stimulated by valinomycin exposure *in vivo*. Yeast strains expressing endogenous Atg11-TAP and/or plasmid encoded Atg11-HA were grown in glucose (*SD*), galactose (Gal), shifted for 2 h from glucose to galactose (Gal2h), treated for 2 h with 4 μM valinomycin (Val), or depleted for nitrogen for 2 h (–N).

We next asked whether mitochondrial damage caused changes in the Atg11 protein. Previously, a starvation-induced interaction of Atg11 with Atg32 at the mitochondrial surface has been shown by bimolecular fluorescence complementation assays (Mao et al., 2013). Since the valinomycin-induced selective mitophagy described here is Atg11 dependent but Atg32 independent, we investigated the intracellular distribution of endogenously expressed Atg11-GFP during the diauxic shift and mitochondrial damage. As shown in **Figure 8A**, Atg11 cannot be detected in yeast cells actively growing in glucose or galactose. After a short induction of mitochondrial damage with valinomycin, we observed the appearance of Atg11 dots close to the mitochondrial network in approximately 35% of the cells. These results suggested that Atg11 concentrates in autophagosomal bodies at the mitochondria rapidly after the induction of damage. Interestingly, the same occurs initially during the diauxic shift, here provoked by the switch from glucose to galactose medium for 2 h. Nitrogen starvation induced mitochondrial Atg11 foci in a smaller number of cells (∼17%). We conclude that valinomycin-mediated mitochondrial damage rapidly induces the formation of Atg11 containing autophagosomal structures at the organelles surface.

Since it is unknown how Atg11 associates with yeast mitochondria upon damage, we performed a two-hybrid screen with Atg11 as the bait upon valinomycin treatment. Among the Atg11 interactors we could not identify a mitochondrial protein candidate, however, we found several clones corresponding to the C-terminal domain of Atg11. We confirmed that a small C-terminal domain (969-1179) was sufficient to interact with the full length Atg11 protein (**Figure 8B**). We then investigated whether the Atg11 dimerization or oligomerization was regulated during the diauxic shift or mitochondrial damage. We therefore performed *in vivo* immunoprecipitation assays using differentially tagged full length Atg11 proteins. As shown in **Figure 8C**, the Atg11-Atg11 interaction was not observed upon glucose or galactose growth, during the diauxic shift or upon short N-starvation. Exclusively after the exposure to valinomycin we observed a clear dimerization of Atg11. These data indicated that Atg11 dimer and/or oligomer formation in the yeast cell is inducible by mitochondrial damage and therefore might represent an early signaling event in the induction of selective mitophagy described here.

## Discussion

Here we report that mitochondrial damage leads to the induction of mitophagy in yeast cells. This result is important because the signaling events underlying mitophagy in yeast have been almost exclusively studied upon different nutrient starvation conditions (Kanki et al., 2015; Abeliovich and Dengjel, 2016). Budding yeast adapts to nutrient availability with a marked regulation of its mitochondrial biomass. Therefore it has served as an excellent model to unravel the molecules involved in the removal of superfluous organelles upon starvation. In comparison, the use of mitochondrial uncouplers in yeast seemed to provoke mitophagy to a much lesser extent (Kissova et al., 2004; Kanki et al., 2009). However, the removal of specifically damaged mitochondria is likely to be mechanistically different from bulk mitochondrial degradation upon starvation, because both processes are triggered by different stimuli. Interestingly, it has been recently reported that inhibition of the electron transport chain by uncouplers induces macroautophagy instead of mitophagy in yeast (Deffieu et al., 2013). This conclusion was based on the apparent lack of the degradation of mitochondrial matrix proteins upon damage, which might be different from the here investigated components of the electron transport chain. Here we apply valinomycin to specifically interfere with the mitochondrial membrane potential. The treatment used throughout our work reduces the respiration rate of yeast cells by approximately 80% and therefore poses a serious threat for cell homeostasis. Under these conditions the appearance of Atg11 containing autophagosomal bodies at mitochondria and subsequent digestion of mitochondrial parts by mitophagy are induced rapidly. It is important to note that a failure in the mitophagic machinery, as shown here for the *atg8, 9 and 11* mutants, causes sensitivity to mitochondrial damage. Thus, mitophagic clearance is a physiologically important repair mechanism upon mitochondrial dysfunction in yeast.

The induction of mitophagy occurs faster in the case of acute damage to the organelle as compared to nutrient deprivation. Thus, a localized mitochondrial depolarization might stimulate autophagosomal formation at the organelle in a much more instantaneous manner. This idea is further supported by the recent demonstration that Atg32 is under strict transcriptional control and that its derepression upon starvation is needed to efficiently engage in mitophagy (Aihara et al., 2014). As compared to this relatively slow process, which relies on de novo synthesis of the Atg32 mitophagy receptor, we show that valinomycin induces mitophagy more rapidly and independently of Atg32. Therefore we propose that yeast cells can induce mitophagic control upon different stimuli using distinct pathways. Interestingly, very recent work has revealed that indeed alternative mitophagy mechanisms exist in yeast (Hughes et al., 2016). Specifically in aged yeast cells a specific subset of mitochondrial membrane proteins is removed by an autophagic mechanism dependent on mitochondrial fission but independent on the Atg32 receptor.

While starvation induces the attachment to mitochondria of Atg11 and the autophagosome via phosphorylated Atg32, we do not know whether a specific mitochondrial receptor other than Atg32 is involved in the valinomycin induced mitophagy. It is likely that a local loss of membrane potential is the initial signal to trigger mitophagy in this case. In this sense it is interesting to note that valinomycin damage rapidly induces the dimerization of the Atg11 adaptor, which might be a mechanism to initiate its accumulation at discrete foci at mitochondria. Under favorable conditions, Atg11 might be actively retained from mitochondria in the cytoplasm by a mechanism, which is overcome by a massive overexpression of Atg11 leading to constitutive mitochondrial localization shown here. Future work will have to investigate whether damage-induced mitophagy in yeast depends on particular mitochondrial receptors. However, it is worth mentioning that the well established Pink/Parkin pathway for the mitophagic removal of damaged mitochondria in mammalian cells seems to rely more on a specific post-translational modification, such as phosphorylated ubiquitin chains, which mark the damaged portion of the organelle, rather than on a specific outer mitochondrial receptor (Herhaus and Dikic, 2015; Lazarou et al., 2015).

An intriguing finding of our work is the preferential degradation of the yeast respiratory complex I equivalent (NADH dehydrogenase) and complex III upon both accelerated respiration and external damage to mitochondria. CI and CIII function might therefore be critically controlled in order to maintain the electron flow throughout the mitochondrial transport chain without incurring in excessive ROS production or lowering the mitochondrial membrane potential. Of note, both complexes are the main producers of ROS in eukaryotic cells (Murphy, 2009; Genova and Lenaz, 2015). Quality control of both complexes upon high respiratory activity seems to rely in great part on the internal proteolysis by mitochondrial proteases. In the light of our results we can assume that if CI and CIII are the “bottlenecks” for the control of the mitochondrial electron transport chain and the maintenance of the mitochondrial membrane potential, the same complexes will be decisive when mitochondrial integrity is challenged by external agents such as valinomycin. Under these more dramatic circumstances, mitophagic removal of CI and CIII might be necessary to maintain mitochondrial homeostasis. In this sense it is important to note that in mammalian cells the Pink1 mitochondrial kinase is dedicated to both the quality control of CI and the induction of mitophagy (Nguyen et al., 2016; Voigt et al., 2016). Accordingly, mutations in Pink1 cause specific defects in CI and CIII activity and abundance (Morais et al., 2009, 2014; Amo et al., 2014). Furthermore, the specific inhibition of CI with rotenone causes the induction of mitophagic repair in mammalian cells, which is critical for cell survival under these conditions (Giordano et al., 2014). Taken together, the performance of specific electron transport complexes is intimately linked to mitophagic quality control mechanisms and our work here in the yeast model suggests that mitophagy is able to selectively remove protein complexes from the damaged organelle. It will be therefore important to decipher the molecular mechanisms that assure this specificity. Importantly, the electron transport chain is known now to organize into several supercomplexes (Acin-Perez and Enriquez, 2014; Genova and Lenaz, 2014). As a consequence, individual respiratory complexes are not randomly distributed in the inner mitochondrial membrane. The advantage of the formation of mitochondrial supercomplexes could be the favored electron transfer from different respiratory subcomplexes to common acceptors such as ubiquinone in the case of CI and CIII and the control of ROS production (Lapuente-Brun et al., 2013; Maranzana et al., 2013; Genova and Lenaz, 2015). In fact the CI/CIII containing complex is one of the most prominent supercomplexes found in higher eukaryotes and yeast Ndi1 CI equivalent has been recently found in complexes with CIII (Matus-Ortega et al., 2015). The supercomplex organization of the mitochondrial electron transport chain could additionally enable the cell to selectively control the quality of modules of the respiratory chain during active respiration or after mitochondrial damage. We identify here mitophagic mechanisms, which seem to act specifically on different mitochondrial protein complexes. It will be therefore crucial to unravel the mechanistic details of this specific mitochondrial quality control in the future.

## Author Contributions

AT-G and DS-R performed all experimental work. AT-G, AP-A, and MP designed the experiments. AT-G, DS-R, AP-A, and MP analyzed the data. AP-A and MP wrote the manuscript. The authors declare that no competing interests exist.

## Conflict of Interest Statement

The authors declare that the research was conducted in the absence of any commercial or financial relationships that could be construed as a potential conflict of interest.
